# Pathogenic *Acanthamoeba* T4 Genotype Isolated from Mucosal Tissue of a Patient with HIV Infection: A Case Report

**Published:** 2017

**Authors:** Fatemeh MEMARI, Maryam NIYYATI, Zeynab JONEIDI

**Affiliations:** 1. Dept. of Parasitology and Mycology, School of Medicine, Shahid Beheshti University of Medical Sciences, Tehran, Iran; 2. Dept. of Medical Biotechnology, Tabriz University of Medical Sciences, Tabriz, Iran; 3. Dept. of Molecular Medicine, Zanjan University of Medical Sciences, Zanjan, Iran

**Keywords:** *Acanthamoeba*, HIV/AIDS, Iran

## Abstract

Opportunistic infections due to free-living amoebae such as Granulomatous Amoebic Encephalitis (GAE), cutaneous acanthamoebiasis and disseminated infections could be the causative agent of mortality in people living with HIV/AIDS. In this study, we report the occurrence of the *Acanthamoeba* belonging to the T4 genotype isolated from nasal and oral swabs of a 15-yr-old man with HIV infection. HIV was confirmed using ELISA kit and RT-PCR assay. The isolated strain showed pathogenic potential using thermo and osmotolerance assays. This patient might be vulnerable to develop GAE or disseminated infections and depending on the immunologic status of the patient, this could be a health threat. Monitoring of such patients, appropriate diagnostic procedures and improved-HIV related care can alter the outcome of such infections.

## Introduction

*Acanthamoeba* spp. are potentially pathogenic free-living amoebae (FLA) successfully isolated from hospital environment, hydrotherapy pools, tap water, soil, sewage and air born dust (1–5). Recent studied showed the occurrence of *Acanthamoeba* in the nasal mucosa and nasopharynx of healthy persons and patients with upper respiratory tract infection (6, 7). Moreover, these amoebae have been isolated from nasal mucosa of immunosuppressed patients such as cancer patients undergoing chemotherapy in Iran (8).

*Acanthamoeba*-related diseases include *Acanthamoeba* Keratitis (AK), Granulomatous Amoebic Encephalitis (GAE), sub-acute granulomatous dermatitis, sinusitis, pneumonitis, and disseminated infections (9–12). Apart from AK, other *Acanthamoeba* pathologies are frequently occur in immunocompromised individuals. Indeed, *Acanthamoeba* could be the causative agent of GAE and disseminated infections, in people with compromised immune systems such as HIV infection, organ transplant, or chronic debilitating illness (13, 14). There are several studies regarding the occurrence of disseminated *Acanthamoeba* infection and GAE in patients with AIDS most of them died due to misdiagnosis (14, 15). Indeed, it is confirmed that *Acanthamoeba* spp. could act as opportunistic pathogens in such patients.

“In the USA, there were about 350 000 deaths because of HIV/AIDS during 1981–1996 with the highest death during the mid-1990s: 49 000 in 1994 and 50 000 in 1995, which declined to 39000 in 1996” (16). Many GAE cases misdiagnose in patients with HIV/AIDS, especially in developing countries. Lack of awareness and misdiagnosis of free-living-amoebae related diseases such as GAE and cutaneous acanthamoebiasis within physician population are the major concern in this regard. This issue is important since early diagnosis of these fatal diseases could alter the clinical outcome (13).

The present study reports the isolation of *Acanthamoeba* T4 genotype from mucosal tissue of a patient with HIV infection using parasitological and molecular method.

## Case report

Here a 15-yr-old man, living in Tehran, Iran in 2015 was admitted to the reference hospital due to severe dyspnea, cough, prolonged fever, chronic diarrhea, shortness of breath and weight loss. The patient was hospitalized in Infectious Ward of a hospital in Tehran for further clinical and laboratory evaluations. Informed consent was taken from the patient.

He had bad taste in mouth, and milky lesions on the inner surface of the mouth. On the tongue and gums as well as candida lesions was observed. In addition, the patient complained of headache, body pain and severe fatigue. Furthermore, lymphadenopathy and adenoid has been reported in his history. HIV antibody test using ELISA kit (Arya Mabna Tashkis Company) was positive. RT-PCR was also used to confirm the result. The CD4 T cell count of this patient was about 300 cell/μl.

**Fig. 1:**
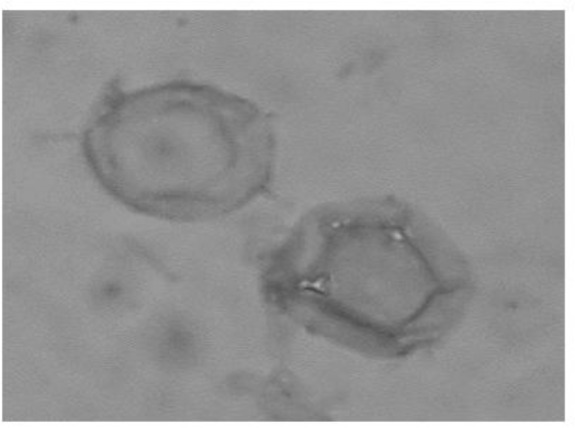
*Acanthamoeba* T4 genotypes isolated from HIV positive patient in Iran

Nasal and oral swabs were taken from the aforementioned patient and cultured in the 1.5% non-nutrient agar (NNA) covered with a layer of heat-killed *Escherichia coli* in Page’s solution. The plate was transferred to the Dept. of Parasitology and Mycology, School of Medicine, Shahid Beheshti University of Medical Sciences, Tehran. The plate was then incubated at 30 °C for to 2 months, and the cultures were checked every day using inverted microscopy. *Acanthamoeba* spp. was detected based on double walled cysts measuring 15 μ (Fig. 1) and flat shaped trophozoites with the unique acanthopodias. In order to achieve a plate without bacterial and fungal contamination several rounds of replicates was performed. Cloned amoebae was harvested from surface of the agar plate using sterile phosphate buffered saline (pH= 7). DNA was extracted using modified phenol-chloroform method (4, 17). PCR reaction was performed using genus-specific primers pairs called JDP1 and JDP2, to amplify a fragment of approximately 500 bp region of the DF3 region in 18S rRNA gene (18). Sequencing of PCR product was performed by the Takapozist Company. To determine the genotypes, sequencing data was aligned with *Acanthamoeba* genotype sequences accessible in the GenBank database. The isolated strain belonged to *Acathaomeba* T4 genotype.

Moreover, thermo-tolerance and osmotolerance test was performed on the cloned strain. Briefly, thermo-tolerance was performed by incubating the strain in two different temperatures (37 and 40 °C). For osmotolerance assay two plates, containing 0.5 and 1.5 M mannitol was prepared and cloned amoebae was passaged. Monitoring of the plates was done during 72 h (19, 20). Interestingly, the isolated amoebae showed high pathogenic potential according the two mentioned tests.

## Discussion

The occurrence of potentially pathogenic *Acanthamoeba* belonging to T4 genotype in mucosal tissue of a HIV+ infected patient may be a concern. This patient was render in developing opportunistic infections such as GAE, cutaneous acanthamoebiasis and disseminated infections due to free-living amoebae. The CD4 T cell count, of this patient was approximately 300 cell/μl. This is significant, as improved-HIV related care for the aforementioned patient, could at least prevent GAE or other complicated infections. As far as we could follow the patient there was no signs of *Acanthamoeba* GAE in the patient. This could be explained by several points as follows: CD4 T cell count of patient was not too low and thus it seems that profound suppress of immune system did not occur at the time of hospitalization. Another probable explanation could be done by differences in pathogenic ability of *Acathamoeba* genotypes as previous studies demonstrated. Indeed, not all isolates of T4 genotype are highly pathogenic (8, 20, 21). These researchers demonstrated that *Acanthamoeba* T4 genotypes isolated from clinical cases has different pathogenic ability.

So far, there is no report of GAE, cutaneous and disseminated acanthamoebiasis in Iran (4), however this is due to the lack of studies and awareness within medical and paramedical population. On the other hand, due to variety of opportunistic infection occurring in AIDS patients, those related to free-living amoebae such as *Acanthamoeba* are usually overlooked (4). Indeed, several protozoan parasites that came into prominence in people living with HIV infection are among potentially pathogenic free-living amoebae including *Acanthamoeba* spp. Unfortunately, there is no effective therapy for the most GAE cases and disseminated infections and thus preventive measures are of utmost importance (12, 15).

Previous researches revealed the presence of *Acanthamoeba* in paraffin-embedded CNS tissue from HIV+ individual with brain lesions using PCR and sequencing based methods (22). The isolated *Acathamoeba* showed a high homology to *A. culbertsoni* in the GenBank. The patient had a very low CD4 T cell count (6 cells/mm^3^) and died due to neurologic disorder within 6 days of hospitalization (22). As mentioned before in the current study the patient had CD4 Tcell (300 cell/ μl) and this may explain the better health situation of the reported case. *Acanthamoeba* related encephalitis was also reported in patients with systemic lupus erythrmatosus confirming the compromised immune statue as pre-requesting factor (23, 24).

There are more than 30 cases of *Acanthamoeba* and HIV co-infection (11). However, the reported rate of *Balamuthia* and HIV is much lower probably due to misdiagnose of amoebae (14). A study showed the occurrence of triple infection *(Balamuthia mandrillaris*, *Acanthamoeba* spp. and *Toxoplasma*) in a patient with advanced HIV infection (14). In addition, the patient with co-infection of HIV and *Acanthamoeba* nodules treated using ketoconazole and 5-fluorocytosine (15). This study reflects that proper treatment could alter the clinical outcome. Moreover, Dowell et al. report a case of AIDS presenting as GAE. The patient was a 41-yr-old male whom *Acanthamoeba* infection was the initial presentation of HIV (25). It is interesting to note that in Europe and the United States GAE is generally linked to AIDS patients (26).

The present study showed that potentially pathogenic *Acanthamoeba* is present in the nasal and oral mucosa of the HIV infected patient. Development of GAE or disseminated infection due to these opportunistic amoebae is probable. The other finding was that the isolated amoebae were belonged to T4 genotype. Tolerance assays revealed that this strain has the pathogenic potential due to growth at high temperature and osmolarity. In vivo assays such as animal based studies are the indicators of pathogenic potential, thus further studies are needed to confirm the pathogenic potential (4, 8).

## Conclusion

The present study highlight further researches regarding the occurrence of free-living amoeba within patients living with HIV/AIDS as such patients are the most susceptible population for developing fatal opportunistic diseases.
